# Surface-Framework structure: A neural network structure for weakening gridding effect in PCB mark-point semantic segmentation

**DOI:** 10.1371/journal.pone.0283809

**Published:** 2023-07-10

**Authors:** Yeshuai Wang, Jianhua Song, Shihui Wang, Yan Zhang, Peng He, Chao Yang

**Affiliations:** 1 School of Computer Science and Information Engineering, Hubei University, Wuhan, Hubei, China; 2 School of Cyber Science and Technology, Hubei University, Wuhan, Hubei, China; 3 Engineering and Technical Research Center of Hubei Province in Educational Informatization, Wuhan, Hubei, China; 4 Engineering and Technical Research Center of Hubei Province in Software Engineering, Wuhan, Hubei, China; 5 Engineering Research Center of Hubei Province in Intelligent Government Affairs and Application of Artificial Intelligence, Wuhan, Hubei, China; University of Alberta, CANADA

## Abstract

Image transfer plays a significant role in the manufacture of PCB; it affects the production speed and quality of the manufacturing process. This study proposes a surface-framework structure, which divides the network into two parts: surface and framework. The surface part does not include subsampling to extract the detailed features of the image, thereby improving the segmentation effect when the computing power requirement is not large. Meanwhile, a semantic segmentation method based on Unet and surface-framework structure, called pure efficient Unet (PE Unet), is proposed. A comparative experiment is conducted on our mark-point dataset (MPRS). The proposed model achieved good results in various metrics. The proposed network’s IoU attained 84.74%, which is 3.15% higher than Unet. The GFLOPs is 34.0 which shows that the network model balances performance and speed. Furthermore, comparative experiments on MPRS, CHASE_DB1, TCGA-LGG datasets for Surface-Framework structure are introduced, the IoU promotion clipped means on these datasets are 2.38%, 4.35% and 0.78% respectively. The Surface-Framework structure can weaken the gridding effect and improve the performance of semantic segmentation network.

## Introduction

Owing to the continuous development of the electronic semiconductor industry, printed circuit board (PCB) manufacturing faces a demand for improvement. Laser direct-writing technology is widely used in the exposure process of PCB manufacturing [1]. In this process, the PCB is positioned and recognized with high precision using machine vision. The position is further transmitted to the exposure device for exposure processing.

The position and recognition of PCBs were usually completed by the mark points on the PCB [2, 3]. The mark point types include holes, laser targets, and other forms [4]. To simplify subsequent process operations and improve recognition accuracy, mark points in the form of circular through-holes were often used in PCB board production [5–7].

In PCB production, a transparent photosensitive dry film is usually applied on the surface of a semi-finished PCB. When a circular through-hole, covered with a photosensitive dry film is captured by the vision system, specular reflection on the surface of the photosensitive dry film often occurs. The shape and size of the reflected light spot formed by the reflection phenomenon varies, and it affects the recognition of the circular mark point.

### Circular hole recognition

The conventional circular hole position method uses the Hough transform algorithm to perform the circular fitting. Another method is to separate the through-hole part from the information of the reflective area, metal, and substrate part, based on the image segmentation of the gray threshold. After segmenting the through-hole area, the contour of the through-hole area was processed using circular fitting. Its stability is better than that of the Hough transform method.

**Fig 1:**
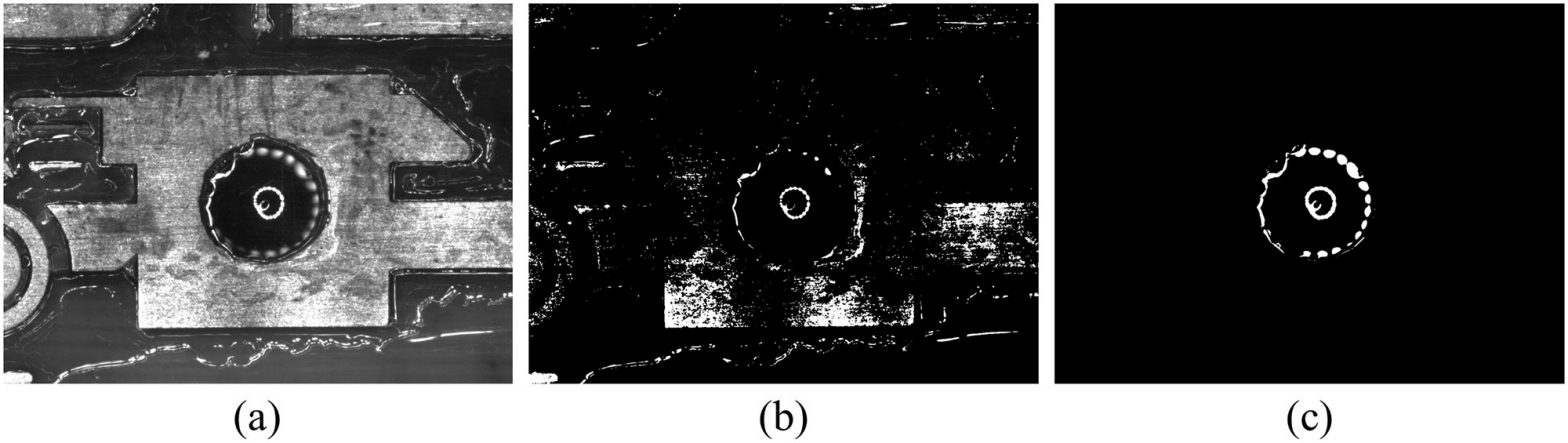
Diagrams of the segmentation method. (a) is mark point image. (b) is the mask w/o ROI. (c) is the mask w/ ROI by automatic labeling.

The segmentation method using gray threshold and manual labeling is shown in Fig 1. When the entire figure was selected for threshold segmentation, the generated mask contained numerous reflective areas outside the through-hole, among which the metal reflective area was large, as shown in Fig 1(b). When the through-hole was considered as the ROI selection range, the threshold segmentation effect was better, as shown in Fig 1(c).

It is crucial to generate a higher-quality mask for image inpainting in the reflection region and improve position accuracy [8, 9]. The quality of the mask generated from the reflective area depended on the environment in which the image was generated. When recognition is unsuccessful, the camera module of the exposure device switches the light source and light intensity, resulting in a significant change in the image’s brightness level. The mask accuracy decreased accordingly. To solve these problems, algorithms designed for different environments consume more time and are less robust. The neural network analyzes image features, can automatically adapt to various production environments, and can be generalized.

To eliminate the influence of reflective spots on image recognition, reflective areas were directly removed or inpainted [10–13]. In this study, specular reflection significantly influenced the processing effect. The inpainting effect was limited by the metal layer near the mark point. Another method is to generate a mask for the reflective part of the image and inpaint the area covered by the mask [14]. The mask is generally generated by traditional image segmentation methods, such as Otsu’s method for gray-threshold segmentation. When the grayscale of the image at the mark point changes significantly, the effect of mask generation is not ideal, and this method has limitations. Mask generation is a typical semantic segmentation task. A semantic segmentation which uses deep learning has a better segmentation effect and stronger adaptability [15–17].

### Semantic segmentation

Semantic segmentation based on deep learning is currently one of the important tasks in computer vision, which has been applied to a variety of scenarios, [18–20].

Based on the low-light environment where reflection changes greatly, many studies on reflection region segmentation based on deep learning have been proposed [21, 22]. In these studies, the image is divided into a reflective layer and a background layer, and the reflective layer generated by reflective objects (glass windows, eyeglasses, etc.) is removed. Since the background layer of Mark points on PCB is unobservable, this method is not applicable.

Semantic segmentation, on the other hand, can be seen as a pixel-dense classification task. Deep learning-based semantic segmentation techniques are based on encoder-decoder (upsampling / deconvolution) structure. It is difficult for convolutional networks to provide enough global features, resulting in insufficient segmentation accuracy. In recent years, researchers have proposed various attention mechanisms to promote global information fusion in the network. However, semantic segmentation networks consume a lot of GPU memory [23].

The neural network structure is gradually becoming more complex, and the attention mechanism (AM) is conducive to the full utilization of data and information and improves the fitting ability of the deep learning network with a small computational cost. SENet [24] and other network structures decompose multiple dimensions of tensors and extract the weight of each sub tensors to enhance the expression of features.

For the network model, Unet based on encoder-decoder is the most commonly used lightweight semantic segmentation network, Most commonly used lightweight segmentation networks, extending the prediction result from one-dimensional semantic information to two-dimensional pixel-wise semantic information [25]. The U-shaped structure based on the encoder-decoder has unavoidable information loss. The skip connection partially compensates for the missing information; however, it lacks sufficient expressive ability. Conversely, a variety of backbones can improve the performance of the network; however, it is difficult to make essential changes to the network. Networks such as Unet and Unet++ [26] promote information sharing between distant modules. The skip connection achieves a better effect at an appropriate location. However, improving the performance of the network is a problem.

Attention Unet [27] redistributed the weight of this stage information to deeper stage information. Feature fusion at different depths can increase the prediction probability of each pixel sample, making the skip connections more adequate [28]. However, this design extracts useless information and is not sufficiently efficient. MultiResUNet [29] provides an effective optimization effect, and the important reason is that the optimization of skip connections can improve network performance.

PCB image has simple semantics, fixed structure, small amount of data, and high precision requirements. Owing to the small number of mark point images containing reflected light spots, it is difficult for the dataset to meet the common large semantic segmentation network structure. Meanwhile, the recognition accuracy of mark points is affected by the quality of mask generation, which requires high performance of the neural network. Unet has the advantages of simple structure, fast calculation speed and good segmentation effect. Models derived from Unet are suitable for PCB manufacturing.

### Gridding effect

Performance is an important requirement of the network model. Gridding effect is an important reason to reduce the performance of semantic segmentation. Gridding Effects are also called Checkerboard Artifacts. Checkerboard artifacts appears when the kernel size is not divisible by the stride [30]. The checkerboard artifacts is fixed when the interpolation method is suitable. However, the semantic loss is inevitable in downsampling-upsampling process.

Dilated convolution enlarges the receptive field of convolution layer without increasing parameters, but introduces gridding effect [30–33]. To reduce the gridding effect, Hybrid Dilated Convolution (HDC) framework [34] is proposed and aggregates global information. The dilation rate of HDC needs to be selected specifically, otherwise the gridding effect will still exist.

Due to the existence of upsampling and deconvolution, gridding effect still affects the segmentation effect. Therefore, how to improve upsampling-deconvolution structure is an important problem in semantic segmentation.

The main contribution of this paper is the proposal of the surface framework structure. And as an example, an improved Unet (PE Unet) based on Surface-Framework structure is proposed. The network model improves the signal-to-noise ratio of the network semantics through pure efficient skip (PES) and multiple stage connection (MSC) modules. The size of the model was designed to adapt to a faster industrial production environment.

The proposed deep learning structure and model will be described in Section “Methods”. The experimental results include some comparative experiments based on different network models and datasets are in Section “Results”, and the main conclusions are in Section “Conclusion”.

## Methods

### Pre-processing

The surface of a prefabricated PCB is usually covered with a copper foil layer with multiple through-hole positions. The surface of the copper foil layer is attached to a photosensitive dry film. After the machine vision system of the exposure equipment initially positions the through-hole, an image of the through-hole is acquired and accurately positioned. To avoid exposure of the photosensitive dry film, the light source used to collect the image is monochromatic such as red light or green light. Therefore, a set of single-channel grayscale images was used to form a dataset of PCB mark points.

We performed data enhancement on 81 mark point images to form a mark point dataset containing 874 images, of which 524 pictures were selected as the training set, and the rest were used as the validation set. The input image size was 1392 × 1040 pixels, which was rescaled to 256 × 256 pixels for training.

The machine vision system only needed to obtain the position of the through-hole area in an image. Therefore, the ROI area was manually set during the generation of the dataset labels. The ROI was selected as the polygonal area at the inner edge of the circular hole, avoiding the reflective area of the copper foil surface. Subsequently, the ROI region image was processed by Otsu’s gray-scale threshold method to generate a ground-truth label, and name the mark point reflective spot (MPRS) dataset, as shown in Fig 1(c).

The pixels number of MPRS data set is from 959 to 21901. The range span is large. Due to the irregular shape and area of the reflective area, the dataset reflects the segmentation effect for different size patterns.

### Surface-Framework structure

The common structure of a semantic segmentation network includes a down-sampling and up-sampling layers. Such a design indirectly increases the receptive field, and down-sampling can significantly reduce the size of the feature map, thus reducing the amount of calculation, causing an irreversible decrease in accuracy, and forming a gridding effect. Therefore, reducing the grid noise caused by the sampling process in the encoder-decoder is essential in semantic segmentation.

**Fig 2:**
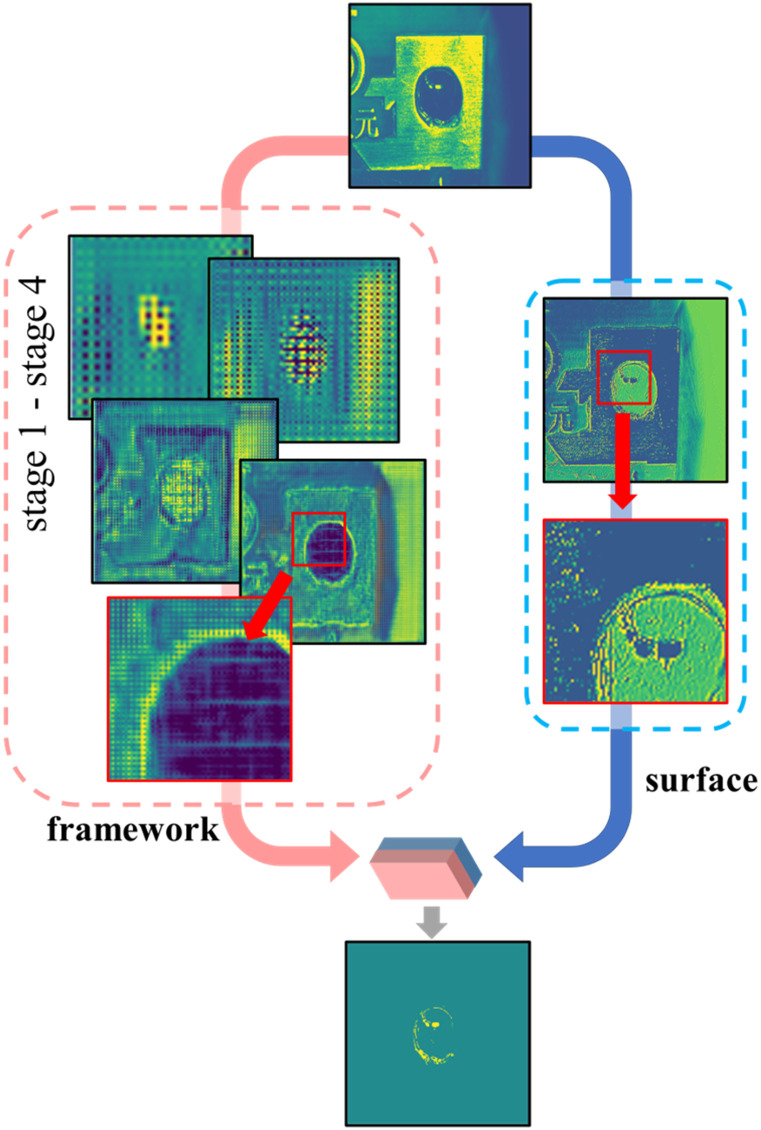
Diagram of Surface-Framework structure.

Using the Unet-like network to perform semantic segmentation on the marked point image has a gridding effect [34, 35], as shown in the green feature maps in Fig 2. It can be observed that the noise information in the deep stage is fused with the surrounding pixels after being processed by the decoder convolutional layer, and its impact on the shallow stage features is reduced. However, the shallow noise information cannot be corrected by the shallower convolutional layer in the decoder. Noise-free image information is required to fuse with the decoder information to improve the integrity of the semantic information.

Therefore, the entire network can be divided into surface and framework parts to form the surface-framework structure. The framework part uses a traditional semantic segmentation network structure to provide deep semantic information to form the basic skeleton of the predicted image. The surface part discards the pooling and up-sampling layers, directly processes the input image with original size, and provides noise-free features. The semantic information provided by the two parts is of the same size and concatenated. During back propagation, the parameters of the two parts can be trained independently without affecting each other. Two parts of the surface and framework can independently become two branches of the network.

The ResPath is proposed in [29], which is used to increase the receptive field at all stages. The top-stage ResPath uses four 3 × 3 convolutional layers and four 1 × 1 convolutional layers. This kinds of network model [29, 36] can be regarded as a form of surface-framework structure.

### Model architecture

**Fig 3:**
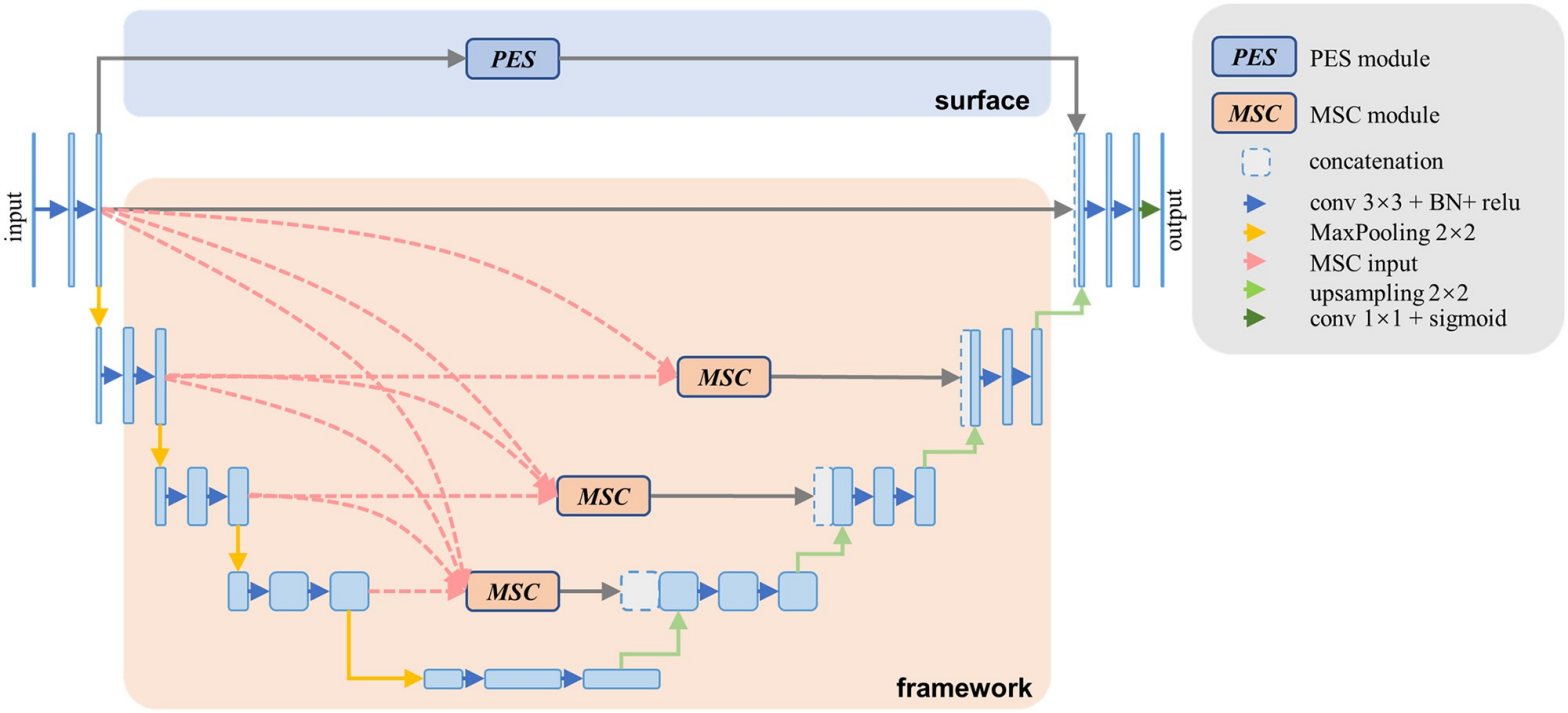
Diagram of the proposed PE Unet architecture.

We propose a segmentation convolutional neural network called pure efficient Unet (PE Unet), as shown in Fig 3. The network was divided into surface and framework parts. The PES and MSC modules are designed in two parts.

For economic reasons, vision systems in automation equipment use low-performance graphics cards or single CPUs for deep-learning calculations. However, the electronic system of the exposure equipment needs to deal with multiple functions; therefore, it has limitations in the use of CPU and memory. Consequently, semantic segmentation using deep learning must play a role in the network performance and hardware resource consumption.

Reducing the number of convolution kernels simplifies the model [37]. For example, the channel number of the first stage in the surface part was set to 16 (a quarter of the original Unet), and the channel number at each stage was doubled compared to the previous stage; therefore, the number of channels at the bottom stage was set to 256. The total parameter was 2.31 M, which is 18.5% of the original Unet.

The loss function uses binary cross entropy (BCE), the convolutional layers of the surface use eight times the first-stage channel in the framework, the convolutional layer channel number of the first stage was set to 16, and in the other stages, it is twice the channel number in the previous stage (unless otherwise specified, these parameters will be used throughout the rest of the paper, every convolutional layer in the PES and MSC modules was connected with a BN layer and a relu layer).

### MSC module

**Fig 4:**
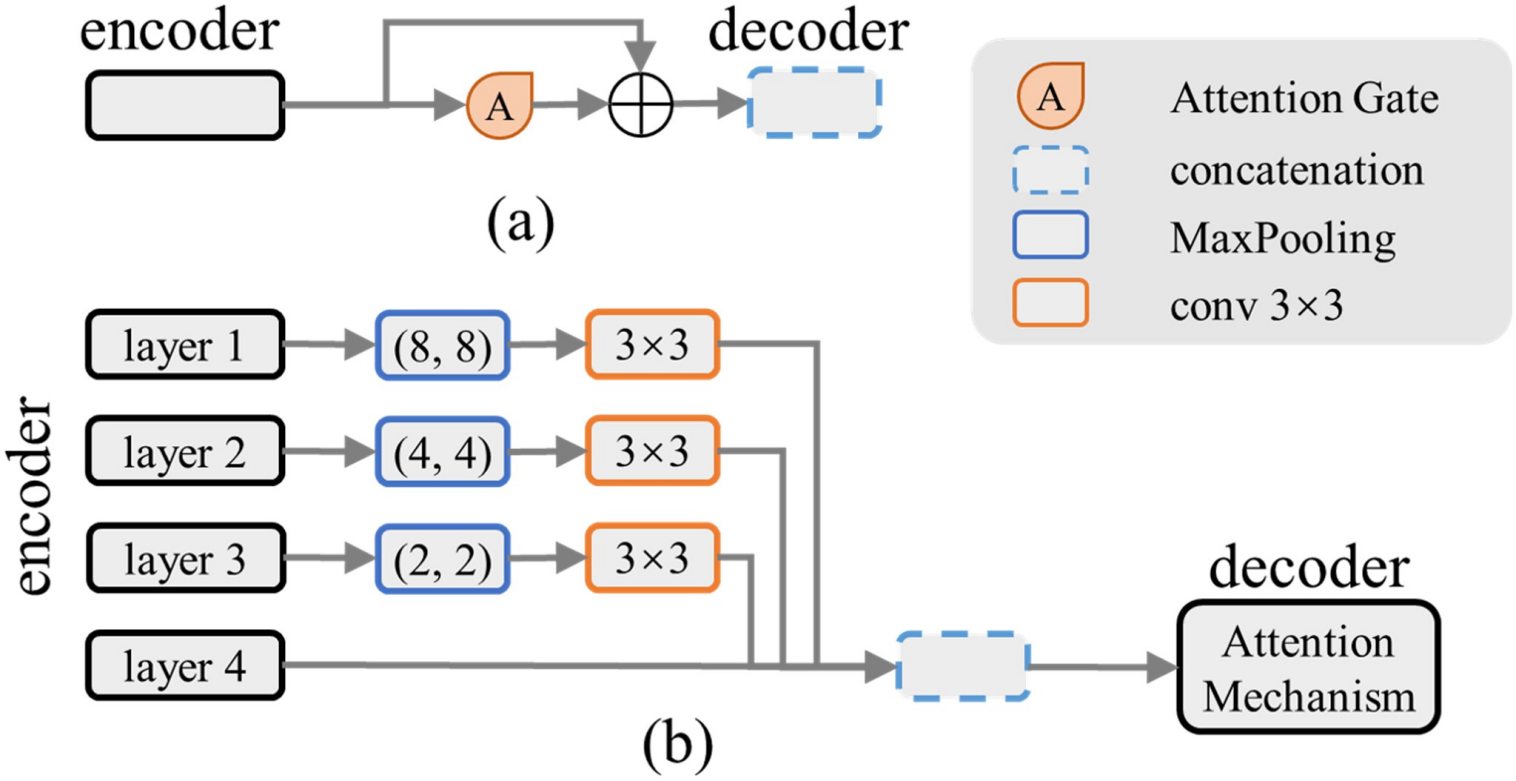
Schematic diagram of MSC module designs.

The AG module of the attention Unet [27] can be regarded as a substitute for the original skip connect, wherein the encoder side is processed in a weighted form, but cannot retain all of its original features. When the number of channels is large, the AG module still has sufficient sparsity to form an invisible skip connection. In the case of fewer features, the performance advantage of the attention Unet over the original Unet is not obvious. Furthermore, a simple skip connection cannot extract the semantic features of the encoder, resulting in a less accurate segmentation effect. We designed a module to reflect the synergy between the AG module and skip connection as case 1 is shown in Fig 4(a). This module is used to enhance the framework Parts.

Conversely, the multiple stage connection (MSC) module is designed to restore the shallow feature details. The module can be placed at a deep stage of the framework. It normalizes and converts certain stage features to the same size and extracts semantic information through the convolutional layer.

In [13], all features of different sizes were merged. In converting deep to shallow stage features, the deconvolution or unpooling layer leaves the noise information of the deep stage features, and the feature information in the decoder is not extracted by sufficient convolutional layers. Therefore, the MSC module does not introduce features deeper than its current stage.

The redundancy of the convolutional layer channel and its sparsity lead to a low efficiency of channel information utilization after the concatenation of multistage features. To improve the utilization of shallow and same stage features, the attention mechanism is used to weigh various multistage features. The MSC module is shown in case 2 in Fig 4(b).

### PES connection

The extraction of feature information is affected by the size of the receptive field. The framework uses downsampling to increase the receptive field. The five stages in Unet realize a receptive range of at least 16 × 16 pixels for a single pixel. The size of the receptive field must match that of the predicted object. The small size and fixed location of medical lesions are the reasons Unet is widely used in medical image semantic segmentation. Similarly, the reflective light spot of the mark point is located in the middle of the mark point, and the size, shape, and position of the light spot are all restricted by the mark point.

**Fig 5:**
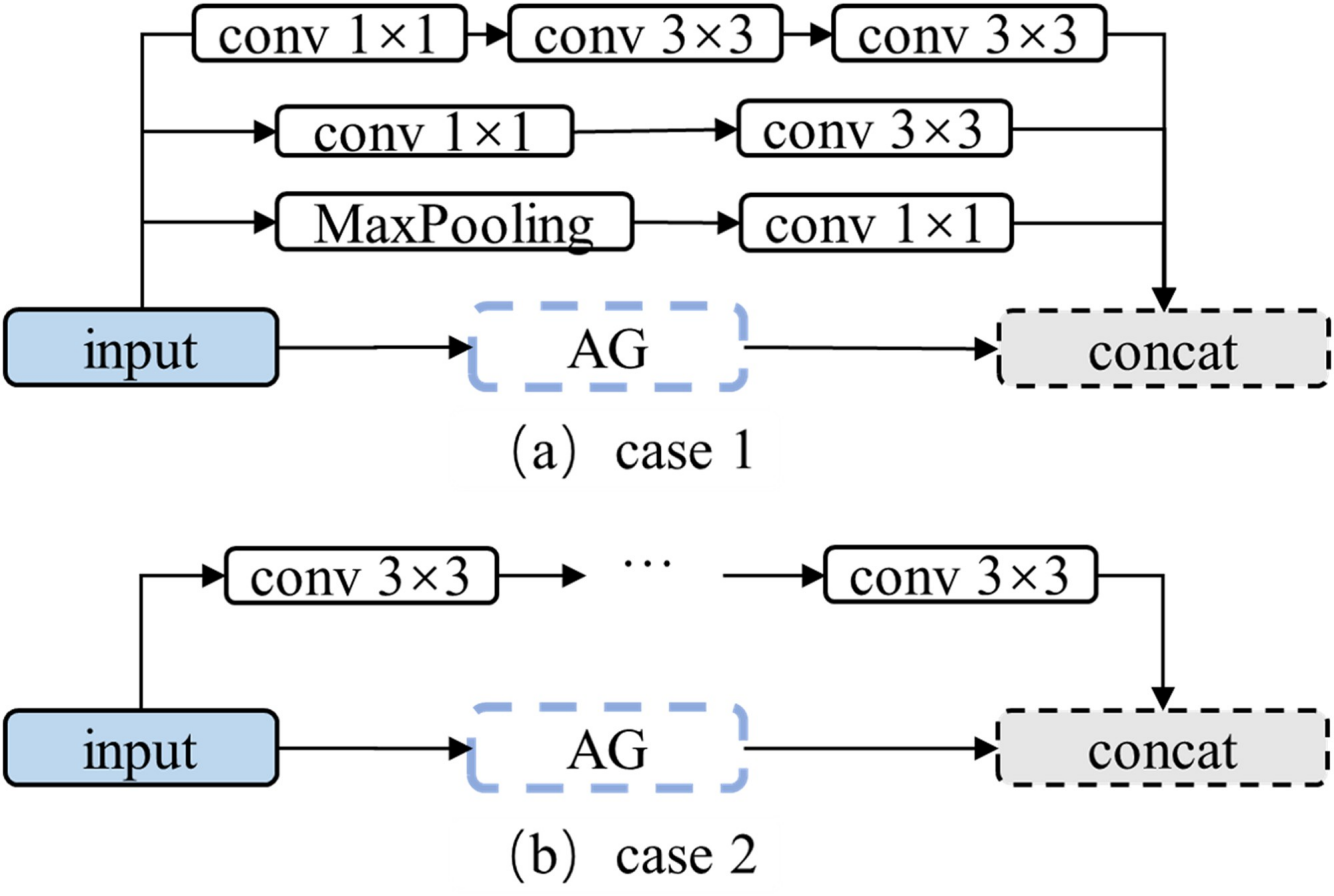
Schematic diagram of PES module designs.

To realize the function of the surface part in our Surface-Framework structure, a pure efficient skip (PES) connection was designed and added to this model between the encoder and decoder as the surface, including two 3 × 3 convolutional layers, skip connection, and the original AG module. A BN layer and a relu layer are added after each convolutional layer. To express the detailed information of the full-size input image, the standard structure of the inception network [38] was tested, as shown in case 1 in Fig 5(a). The MaxPooling layer may filter out detailed information and weaken the effect of the detailed expression; therefore, it is removed, as shown in case 2 of Fig 5(b). The AG module was added to these structures to enhance the feature details. The gating signal of the AG module is from decoder stage 2 of the framework, which may introduce some gridding effect noise. However, the IoU is improved after the AG module is added, and its advantages are dominant. Moreover, owing to the source of the gating signal, the AG module can be regarded as a framework part instead of a surface part.

### Back propagation

Generally, metrics such as IoU and DICE are used to evaluate the network performance. In the early stages of network training, some gradient disappearance phenomena appear in these evaluation metrics. In the concatenation process, the weight parameters of each feature channel are gathered. Suppose w is the weight parameter and L is the loss function, the back propagation can be written as

$$\begin{array}{c} \frac{\partial L}{\partial w}=\frac{\partial L}{\partial z}\cdot \frac{\partial z}{\partial w} \end{array}$$ (1)

note

$$\begin{array}{c} z=\sum_{i=1}^{n}\left(w_{i}\cdot a_{i}+b_{i}\right) \end{array}$$ (2)

we have

$$\begin{array}{cc} \frac{\partial z}{\partial w} & =\frac{\partial (\sum_{i=1}^{n}\left(w_{i}\cdot a_{i}+b_{i})\right)}{\partial (\sum_{i=1}^{n}w_{i})}\\ & =\sum_{i=1}^{n}\frac{\partial (\sum_{i=1}^{n}\left(w_{i}\cdot a_{i}+b_{i})\right)}{\partial w_{i}}\cdot \frac{\partial w_{i}}{\partial (\sum_{i=1}^{n}w_{i})}\\ & =\sum_{i=1}^{n}a_{i}/(1+\frac{1}{w_{i}}\cdot \sum_{1\leq j\leq n,j\neq i}^{}w_{j}) \end{array}$$ (3)

In the case of fewer weight parameters, when w_*i*_ is zero, $\frac{\partial z}{\partial w}$ approaches zero, and the gradient disappears. Therefore, reducing the number of channels has a negative effect on the training process.

## Experiments and results

All experiments were completed using TensorFlow 2.3, the optimizer was Adam, the initial learning rate was 0.001, and the batch size was set to 4. The hardware used a GPU server (CPU is Intel Xeon Silver4210 @2.2 GHz * 2, memory of 24 GB, GPU NVIDIA Quadro RTX 5000, GPU memory 16 GB).

### Evaluration metrics

To demonstrate the advantages of the proposed method in terms of speed and precision, we compared a variety of common segmentation models (Unet [25], R2U-Net [39], attention Unet [27], Res Unet, Unet++ [26], Segnet [40], ours). The evaluation metrics selected were IoU, precision, recall, and F1 score. The effect of using Surface-Framework structure in each network is also compared. We form the simplest surface part with two convolutional layers (each convolutional layer is connected with a BN layer and a relu layer, each convolutional layer’s channel number is 8 times that of stage 1 channel number). The evaluation performance results are presented in Table 1.

**Table 1:** Results of comparative experiments with different metrics.

Method	stage 1 channel number	IoU (%)	Precision	Recall	F1 Score	Parameters (M)*	Time (ms)*
Unet [25]	16	81.59	90.48	88.90	88.41	1.95 / 2.13	69 / 148
R2U-Net [39]	16	81.33	91.15	89.85	88.24	6.39 / 6.56	136 / 220
Attention Unet [27]	16	81.54	91.04	89.62	88.44	2.01 / 2.20	81 / 163
ResUnet [41]	32	81.37	90.39	89.96	88.02	18.85 / 19.69	268 / 585
Unet++ [26]	32	80.24	92.67	86.83	87.99	9.06 / 9.80	270 / 544
Segnet [40]	32	71.01	85.20	76.28	80.38	7.37 / 8.09	164 / 400
Ours	16	84.74	91.84	90.89	90.37	- / 2.31	- / 180

* The data before and after symbol “/” are respectively w/o and w/ Surface-Framework structure

The proposed method achieved good results for a small parameter. Compared to other network models, it exhibited an obvious improvement in most metrics. When the number of channels is small, the vanishing gradient problem often occurs in the training process of the R2U-Net, and the number of parameters is significantly higher than those in other networks. The performance of R2U-Net is close to that of Unet and attention Unet.

ResUnet with a residual block is an improved version of the Unet. We chose Resnet 18 as the backbone of ResUnet [41]. For small parameters, ResUnet performed poorly. When the number of stage 1 channels is set to 64, the IoU of ResUnet reached 82.65%, which is better than that of Unet. We believe that the fullness of the channel number conceals the inefficiency of simply adding the residual connections.

Unet++ cascaded multiple Unets of different depths to adapt to an appropriate network depth with different receptive fields. The number of stage 1 channels of the original Unet++ is 32, and its IoU performance is poor. In addition to the precision metric, other metrics were also low.

The hardware environment for time-consuming testing is a PC with an Intel Core i7-9700 CPU and 16 GB memory. After adding the Surface-Framework structure, the predicted time under different network structures is from 148 ms to 585 ms. In general, the predicted speed can meet the needs of industrial production.

### Structure pruning

#### MSC module

Based on MSC module design as shown in Fig 4, an ablation experiment is conducted, and the experimental results are presented in Table 2. Our MSC Module improves network performance. The fusion of Multi-stage feature plays an important role in the module.

**Table 2:** Ablation experiment of MSC module for MPRS data set.

Method	Multi-stage feature	AM	IoU (%)
Att Unet + surface part	None	None	84.13
case 1	None	None	84.44
case 2	Yes	None	84.51
case 2	Yes	AG	84.37
case 2 (ours)	Yes	SE	84.74

#### PES module

Regardless of the detailed feature information, when the deep-level structure feature information of the reflective spot is less, the fourth and fifth stages of the network structure have less impact on the network performance than on other stages.

From the first stage to the fifth stage of the framework, we took 1/4 times the channel number of the convolutional layer for comparison experiments. The IoU is 83.34%, 84.38%, 84.54%, 84.68%, and 84.79% in sequence. The experimental results are listed in Table 3. The experimental data show that the main source of prediction errors is the details in the image rather than the image structure.

**Table 3:** Results of comparative experiments under 1/4 convolutional layer channel number.

Channel number of the convolutional layer					IoU (%)
stage 1	stage 2	stage 3	stage 4	stage 5	
4	32	64	128	256	83.34
16	8	64	128	256	84.38
16	32	16	128	256	84.54
16	32	64	32	256	84.68
16	32	64	128	64	84.79

In the case of numerous convolutional layers in the PES module, the difference between the encoder and decoder features is large, resulting in a decrease in network performance. Several structures were designed for comparison, and the experimental results are listed in Table 4.

**Table 4:** Ablation experiment of PES module for MPRS data set.

Surface part method	conv number	IoU (%)	Parameters (M)
case 1	/	83.88	3.28
case 1 w/o MaxPooling	/	85.02	3.27
case 2	1	83.09	2.51
	2 (ours)	84.74	2.31
	3	84.63	2.59
	4	83.31	2.74
case 2 w/o conv	/	82.03	2.11
case 2 w/o AG	2	84.26	2.31

It is evident that inception-like networks can predict the image details well. However, a simple series of convolutional layers can achieve similar effects. The number of series of convolutional layers should be determined according to the size of the receptive field required to express detailed information. Moreover, a large number of convolutional layers will increase the network complexity and computing time.

Furthermore, the addition of PES connections to the framework does not enhance network performance, and the number of PES convolutional layers is weakly correlated with network performance.

### Channel pruning

When the network structure is determined, the number of convolutional layers is an important factor that affects the image segmentation quality. The automatic label generation method of image preprocessing reduces noise in the features. Simultaneously, the number of deep-stage structure features in the framework is limited, and the predicted object size is positively correlated to the receptive field size. Therefore, less information is needed to fully express the image features, and the channel number of the feature information is reduced accordingly.

Because the smaller size of the deep stage features does not require considerable computing power, the network retains the setting of the convolutional layer channel number in the backbone of the framework. In addition, increasing the convolutional layer channel number of the PES and MSC modules had no effect on the network performance. Assume that the MSC module reduces the l-th to the m-th stage feature size by the MaxPooling layer and uses the convolutional layer C_*n*_ for feature extraction. The channel number of the convolutional layer C_*n*_ was set to four times the channel number of the m-th convolutional layer. The convolutional layer in the series of the PES module in the surface part uses eight times the size of the stage 1 convolutional layer.

To clarify the effect of network design on different parameters, an ablation experiment was designed, as shown in Table 5. When the convolutional layer channel numbers at stage 1 are 8, 16, 32, and 64, the network is 2.76%, 3.15%, 2.81%, and 2.75% respectively, which is higher than that of Unet. The doubled increase in the number of channels resulted in a slight improvement in the performance. When the number of stage 1 channels reached 16, IoU reached 84.74%. When the number of stage 1 channels is 32, the parameter amount increased by 300%, and the IoU only increased by 0.61%.

**Table 5:** Ablation experiment of PE Unet for MPRS data set.

method	IoU (%) when stage 1 channel number is:			
	8	16	32	64
Unet	80.02±0.69	81.59±0.53	82.54±0.32	82.52±0.54
Unet + MSC	79.88±0.69	81.88±0.47	83.01±0.42	83.28±0.43
Unet + PES	82.70±0.60	84.22±0.38	84.92±0.39	84.89±0.52
PE Unet	82.78±0.56	84.74±0.58	85.35±0.41	85.27±0.53

To further clarify the impact of the increase in the number of channels on the computing power requirements, we conducted tests under different computer hardware configurations, as shown in Table 6. We chose three test environments to represent high-, middle-, and low-level hardware configurations. The first environment is our GPU server; the second environment is a desktop computer without a graphics card, CPU is Intel Core i7-9700 @ 3.00 GHz, memory is 16 GB; the third environment is a desktop computer without a graphics card, CPU is Intel Core i5-4200H @ 3.40 GHz, and memory is 8 GB. When the number of stage 1 channels is 16, it takes 169 ms without a GPU, which can meet the tempo requirements of high-speed production on the production line.

**Table 6:** Results of network computing resource consumption in different hardware environments.

stage 1 channel number	Parameters (M)	Prediction time (ms)			Memory	GFLOPs
		Environment 1	Environment 2	Environment 3		
8	0.58	26	89	268	254	8.5
16	2,31	28	180	487	512	34.0
32	9.24	36	462	1335	1040	135.6
64	36.91	73	1310	4537	2149	541.6

### Comparative experiments of Surface-Framework structure

**Fig 6:**
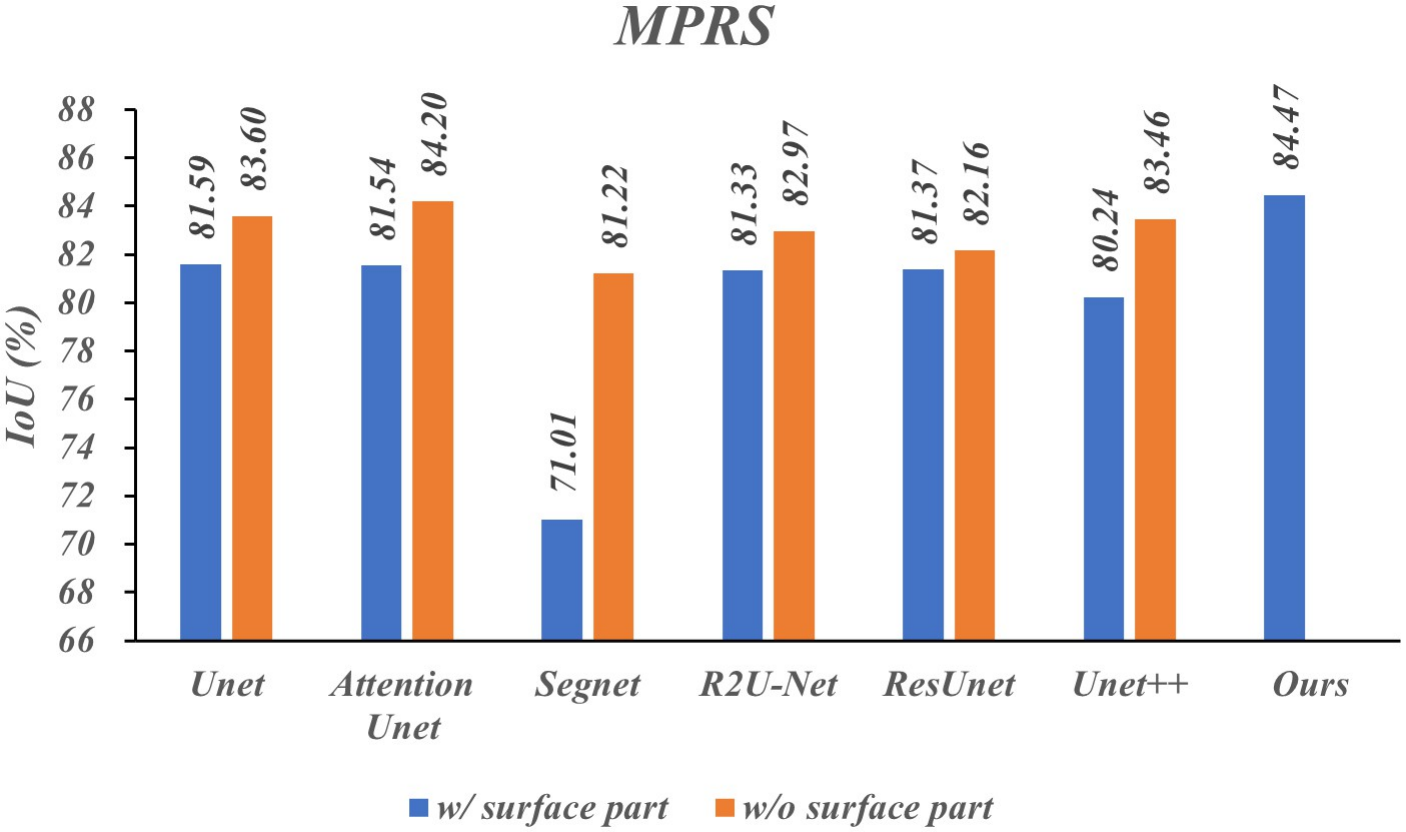
Results of comparative experiments under different segmentation models on MPRS dataset.

**Fig 7:**
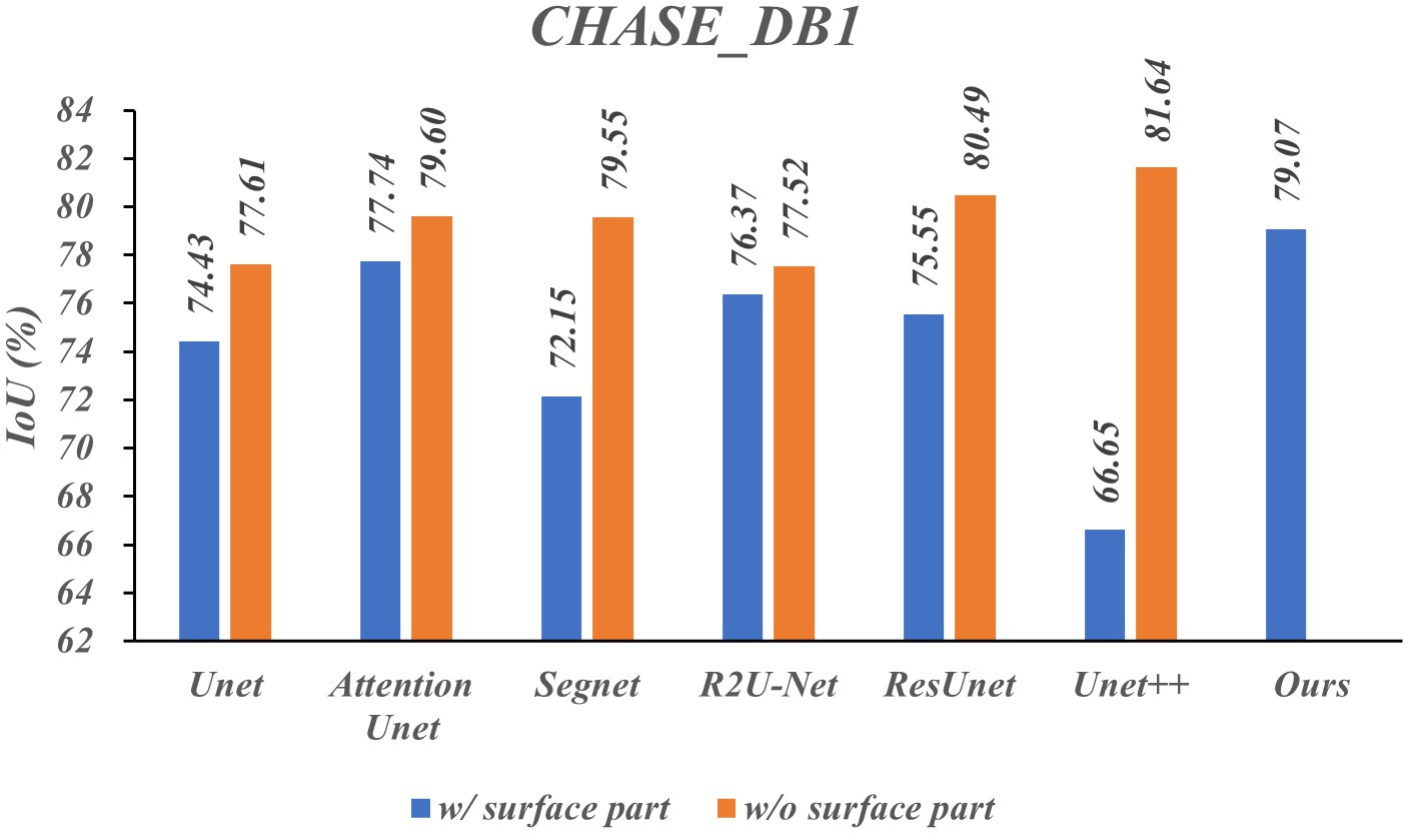
Results of comparative experiments under different segmentation models on CHASE_DB1 dataset.

**Fig 8:**
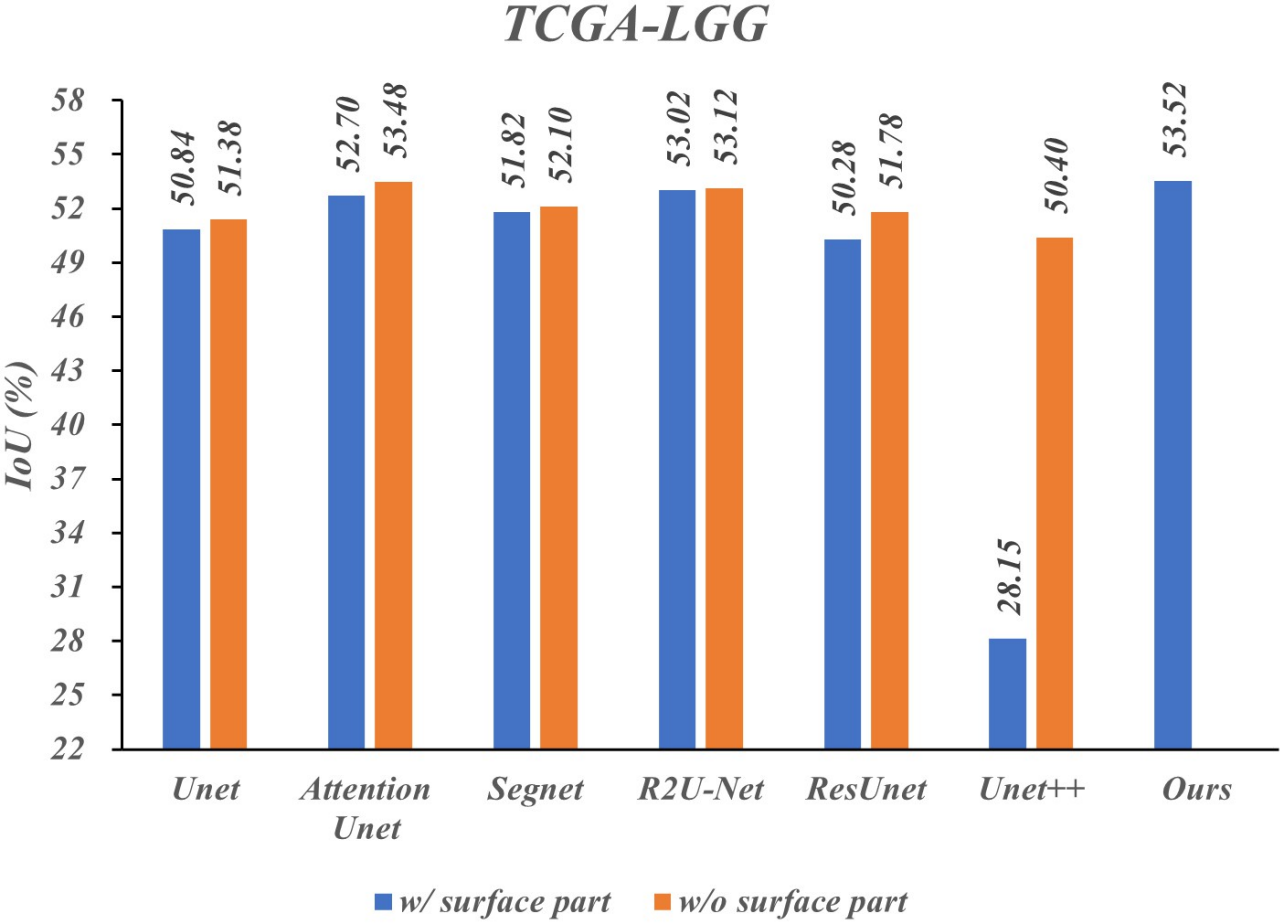
Results of comparative experiments under different segmentation models on TCGA-LGG dataset.

To verify the generalization of the Surface-Framework structure, we designed several comparative experiments with additional two public datasets, CHASE_DB1 [42] and TCGA-LGG (the dataset is publicly available and released at the following link: https://www.kaggle.com/mateuszbuda/lgg-mri-segmentation). The MPRS and CHASE_DB1 datasets were enhanced by moving, rotating, mirror flipping and so on. The TCGA-LGG dataset was not enhanced due to the large number of dataset. The IoU w/ and w/o surface part were measured on above three datasets, respectively. The results are presented in Figs 6–8 respectively.

As shown in Fig 6, SegNet performs poorly on our MPRS dataset. However, the IoU is significantly increased with adding a surface part. In general, the IoUs were improved from 0.79% to 10.21% with adding a surface part. The IoU promotion clipped mean w/o maximum and minimum value is 2.38%.

As shown in Figs 7 and 8, Unet++ performs poorly on these datasets. Especially on the TCGA-LGG dataset, the IoU with Unet++ is only 28.15. The possible reason is that the TCGA-LGG image size is fairly small (256 × 256), causes the error introduced by the downsampling layers is relatively large, which makes the network performance worse. And the surface part significantly improves the performance of the Unet++. The IoU promotion clipped means w/o maximum and minimum value on CHASE_DB1 and TCGA-LGG were 4.35% and 0.78% respectively.

### Segmentation result

**Fig 9:**
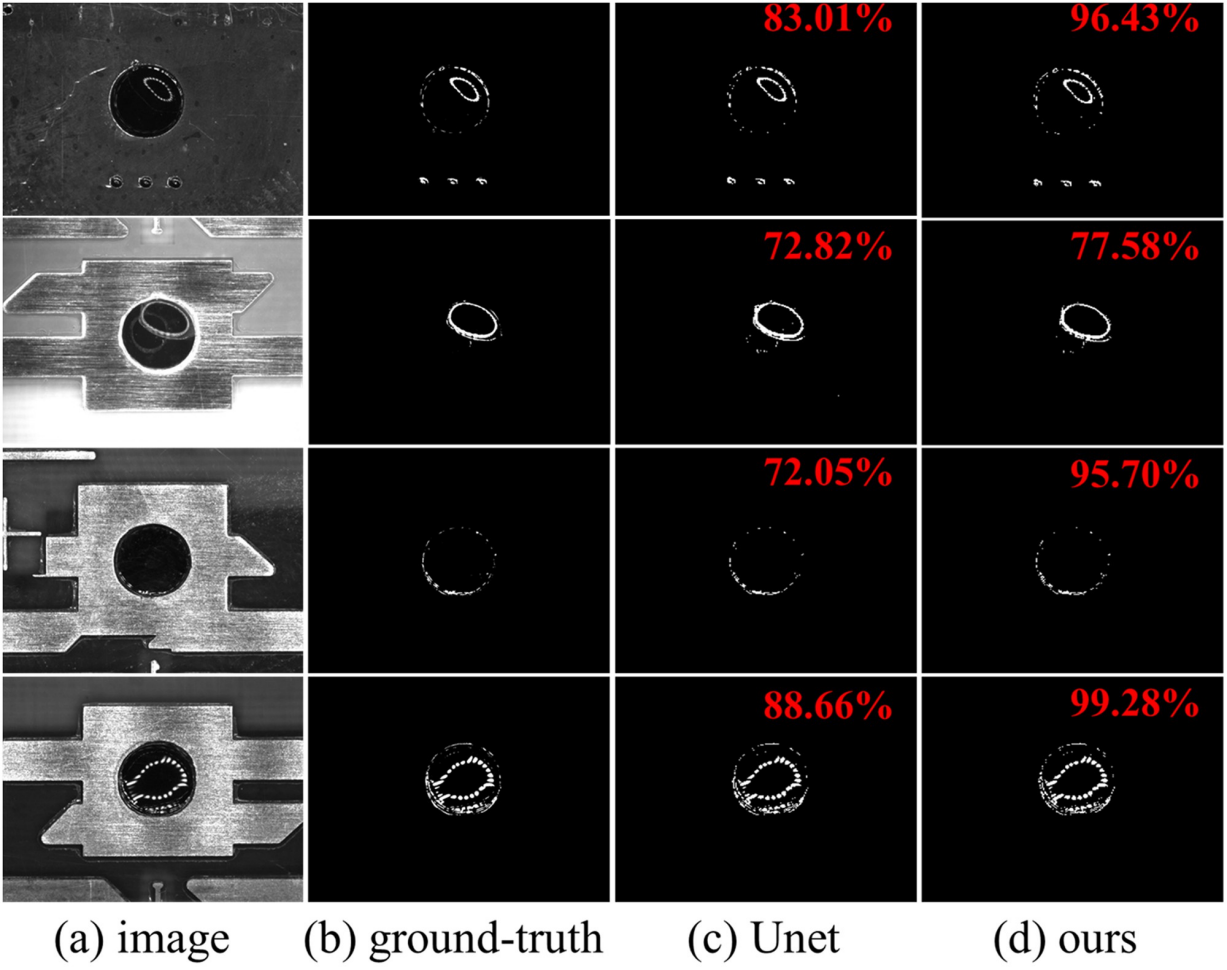
Qualitative comparison between Unet and PE Unet. Segmentation results for our four distinct mark point image segmentation applications. The red words in the figure are the prediction results using the IoU metric. (a) image (b) ground-truth (c) Unet (d) ours.

**Fig 10:**
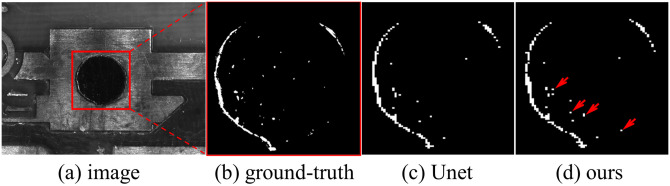
Comparison of segmentation details between Unet and PE Unet. The red arrows in (d) were the pixel of the positive sample not recognized by Unet. (a) image (b) ground-truth (c) Unet (d) ours.

The results are shown in Fig 9. The difference between the results using Unet and PE Unet is greater in the local pixel prediction than in the overall framework, as shown in Fig 10.

**Fig 11:**
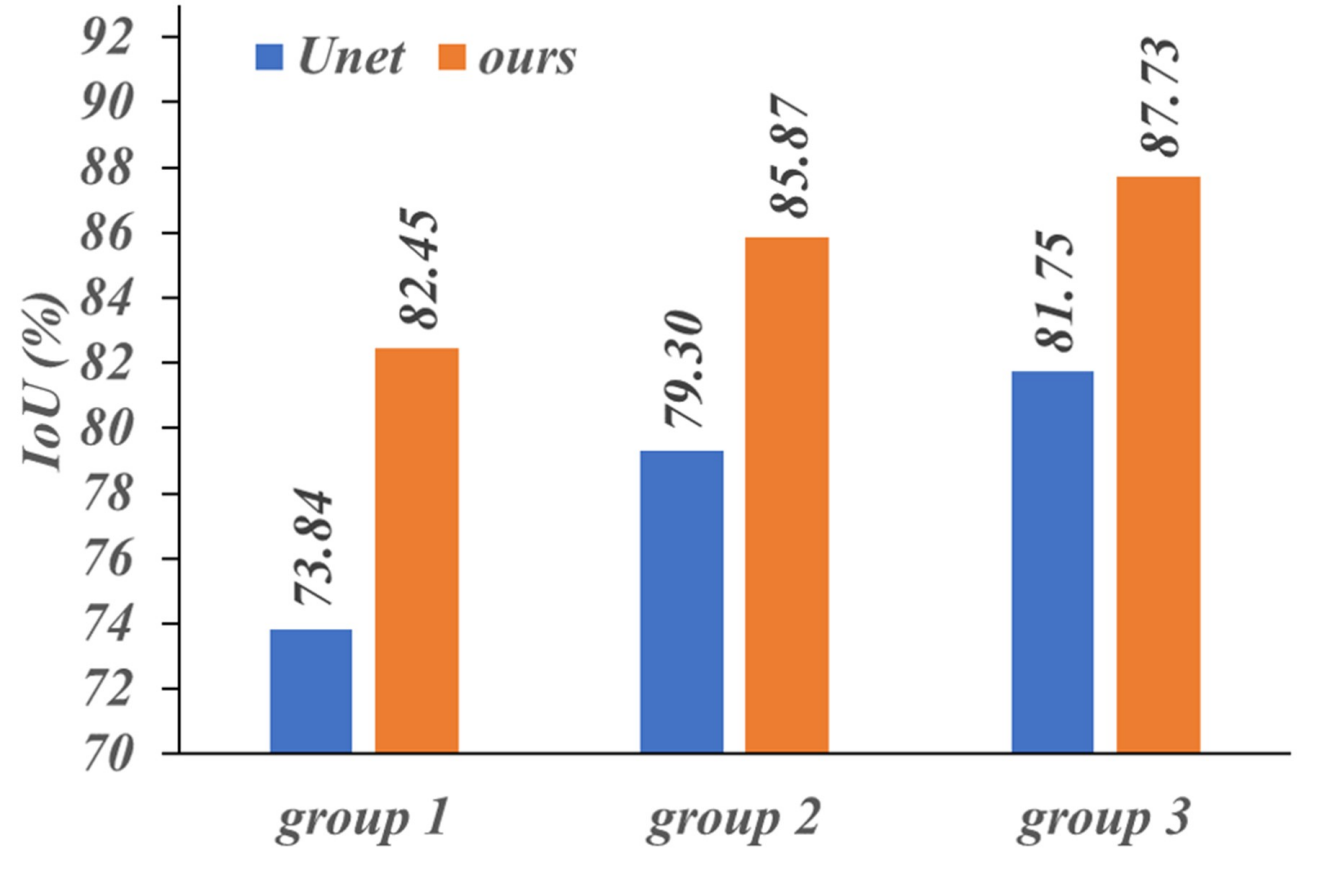
Results of the segmentation effect when the data set is divided into three groups.

The MPRS dataset was divided into three groups according to the number of pixels in the reflective area, from small to large. The ranges of the pixel numbers in the three groups of images were 959-4852, 4852-7986, and 7986-21901 respectively. Fig 11 shows three groups of image segmentation results. The IoU, using PE Unet is 8.61%, 6.57%, and 5.98% higher than that which uses Unet. PE Unet performed better than Unet in terms of segmentation with more details.

## Conclusion

We have presented a Surface-Framework structure. And an extension of the Unet model (PE Unet) using the Surface-Framework structure is proposed. The improved performance by our PE Unet is attributed to its surface part design w/o framework part noise and multiple stage connection. The proposed modules are called “PES module” and “MSC module”. Surface-Framework structure in PES module provides noise-free detail features. The shallow noise-free features from surface part compensate for the error generated by deep semantic features from framework part, thereby weakening the grid effect. Our model and structure are evaluated using three different applications in the field of PCB reflective spot segmentation, retina vessel segmentation and brain segmentation. Several comparative experiments based on various existing models and all three datasets are introduced. The visualize and quantitative results demonstrate that simply using our Surface-Framework structure can effectively improve the existing network segmentation performance. The proposed PE Unet, as a case of our Surface-Framework structure, also shows good performance in the experiments.

The existing literature and data show that the optimal network structure is closely related to the characteristics of different datasets. A common approach is to exhaust the existing network structure. How to understand the pattern features is the key to explain the differences in network performance. In the future, we will explore how the shape and size of the dataset affect segmentation performance, so as to further explore the mechanism.
